# Resistance and cross-resistance in populations of the leafrollers, Choristoneura rosaceana and Pandemis pyrusana, in Washington apples

**DOI:** 10.1673/2006_06_14.1

**Published:** 2006-08-25

**Authors:** John E. Dunley, Jay F. Brunner, Michael D. Doerr, E. H. Beers

**Affiliations:** Washington State University, Tree Fruit Research and Extension Center, 1100 N. Western Ave., Wenatchee, WA 98801

**Keywords:** insecticide resistance, organophosphate, insect growth regulator, benzoylhydrazine, LCR Lethal concentration ratio, LC_50_ Lethal concentration (50% of the population)

## Abstract

Insecticide bioassays of the leafrollers, Choristoneura rosaceana (Harris), and Pandemis pyrusana Kearfott (Lepidoptera: Tortricidae), were used to investigate resistance and cross-resistance between azinphosmethyl and other insecticides. Comparisons of field-collected populations with susceptible laboratory colonies of both leafroller species were made in 1996–97, prior to registration and field introduction of several of insecticides, and were re-tested in 2000–2001 following several years of use in the field. Insecticides tested included azinphosmethyl, chlorpyrifos, methyl parathion, tebufenozide, methoxyfenozide, spinosad, indoxacarb, acetamiprid, Bacillus thuringiensis, and azadirachtin. Azinphosmethyl-susceptible laboratory colonies were used for comparison to field populations. Resistance to azinphosmethyl was found in all populations of C. rosaceana (5.2–26.8 fold) and P. pyrusana (8.4–24.9 fold) collected from commercial orchards. Cross-resistance between azinphosmethyl and the insect growth regulators tebufenozide and methoxyfenozide was found in all but one population of the two leafroller species. No cross-resistance was found to chlorpyrifos. Some of the populations tested were cross-resistant to spinosad and indoxacarb, but the responses to these materials were more variable.

## Introduction

Leafrollers are among the most destructive lepidopteran pests of tree fruits in Washington, second only to codling moth, Cydia pomonella. Although a number of leafroller species occurs in Washington, two species,Choristoneura rosaceana (Harris), and Pandemis pyrusana Kearfott, are the most important in commercial orchards. Damage from larval feeding on fruit can occur around bloom, during mid-summer, and just prior to harvest (Beers *et al.* 1993).

Leafrollers, and a wide spectrum of other apple pests, have been controlled using the organophosphate insecticides for over four decades. Organophosphate insecticides have played a key role in pest management during that time, especially after the phase-out of organochlorine insecticides. Organophosphate insecticides initially had a very wide spectrum of activity, including control of most primary and secondary insect pests of apple, as well as tetranychid mites. Efficacy against various pests declined over time with greater or less rapidity, until lepidopteran pests were the primary remaining targets. By the mid-l990s, only a few organophosphate insecticides were still widely used for codling moth and leafroller control, including azinphosmethyl, chlorpyrifos, and methyl parathion (Beers *et al.* 1996).

The organophosphate-based apple integrated pest management system was relatively stable for many years; however, regulatory action on organophosphate insecticides (Food Quality Protection Act of 1996) brought pressure for change in the fundamental approach to apple integrated pest management. In addition, there has been increasing evidence for organophosphate resistance in the codling moth, a key pest (Dunley and Welter 2000;Knight *et al.* 1994; Varela *et al.* 1993), along with reports of decreasing efficacy against leafrollers (Brunner 1996). The introduction of pheromone mating disruption represented a major change in apple integrated pest management and resulted in significant reductions in the use of organophosphate insecticides (Thomson *et al.* 2001, Brunner *et al.* 2002). The successful implementation of mating disruption of codling moth has promoted the investigation of alternative chemistries for lepidopteran pest control that are potentially less toxic to natural enemies, including Bacillus thuringiensis, insect growth regulators, neem-based insecticides, neonicotinyls, and fermentation products. This change in approach has greatly diversified the array of control tactics available to the producer, and has provided greater opportunities for biological control.

The purpose of this study was to determine the status of susceptibility of the two primary leafroller pests to organophosphate insecticides, and examine field populations of leafrollers for resistance and cross-resistance to new insecticides.

## Material and Methods

### Rearing and Bioassay Methods

Laboratory colonies of C. rosaceana and P. pyrusana were collected initially from field populations occurring on apple. The P. pyrusana colony was collected from Yakima, WA, in 1985, and the C. rosaceana colony was collected from Mattawa, WA, in 1990. These two colonies have been reared continuously since their collection on a pinto bean diet using the method of Shorey and Hale (1965) under constant growth room conditions of 23 ± 2°C, with a photoperiod of16:8 L:D.

C. rosaceana and P. pyrusana field populations were collected either from the overwintering or summer generation larvae. Larvae were reared on pinto bean diet in the laboratory through to neonates of the first generation. Field populations were collected from two apple blocks in the Tree Fruit Research and Extension Center in Wenatchee Washington (TF1 and TF2); the Wenatchee Valley College orchard in East Wenatchee (WV); Stemilt Hill (SH), south of Wenatchee, on the west side of the Columbia River; Columbia River Orchards (CRO), 20 miles south of Wenatchee on the east side of the Columbia River; Quincy Washington (QC); Mattawa Washington (MA1 and MA2); Brewster Washington (BR); Bridgeport Washington (AR); an orchard on the Snake River, near Pasco, Washington (BO); and Milton-Freewater, Oregon (MF1, MF2, and MF3).

In 2000–01, more populations from a wider geographic distribution were tested. Three products tested in 1996–97 were not retested because of changes in use. Chlorpyrifos use in apple had been restricted to the pre-bloom period, methyl parathion registration had been withdrawn completely, and tebufenozide use had been largely replaced by methoxyfenozide. Indoxacarb, a newer material in the registration process, was added.

A leaf disk bioassay was used to expose leafroller larvae to insecticide residues. Treatments (insecticide concentrations) were prepared by dilution of the formulated insecticide in 500 ml water. A wetting agent, Latron B-1956 (Dow AgroSciences, www.dowagro.com/) (2 ml) was added to the stock solution. Serial dilutions were made from the stock solution, with 4 to 8 concentrations per insecticide. The control was water plus the wetting agent.

Leaves were collected from untreated apple trees (Malus domestica Borkhausen) at the Washington State University Tree Fruit Research and Extension Center, Wenatchee. Whole leaves were dipped in the various insecticide concentrations and allowed to air dry. Disks (2.3 cm diameter) were then cut from each leaf, and four leaf disks were placed in a small covered Petri dish (Falcon 1006, 50 x 9 mm, Becton-Dickinson Labware,www.bd.com/). Five 1- to 2-day-old leafroller larvae were placed at random on the leaf disks in each dish. A total of 10 dishes (50 larvae) were prepared per insecticide concentration. Petri dishes were placed in a plastic container and held at 23 ± 2°C and 16:8 (L:D). Larvae were examined after 7 d and mortality recorded, except for the two azadirachtin compounds, for which mortality was recorded after 14 days. Failure to respond to gentle probing with a camel's hair brush was classified as dead.

### Statistical Analysis

Probit regression parameters (slope and LC) were estimated using the probit option of POLO-PC (LeOra 1987). Significant differences in LC_50_ between leafroller species were determined by non-overlapping 95% fiducial limits. Comparisons with susceptible populations among leafroller species were made using a lethal concentration ratio tests (LCR) (Robertson and Preisler 1992); ratios that had confidence limits not encompassing 1.0 were considered significantly different (α = 0.05).

## Results

The two laboratory colonies of C. rosaceana and P. pyrusana did not differ significantly in their responses to the organophosphate insecticides azinphosmethyl and methyl parathion, althoughP. pyrusana was more susceptible to chlorpyrifos thanC. rosaceana (Table 1). There were no differences between the species for the insecticides with insect growth regulator activity. P. pyrusana was more susceptible to indoxacarb than C. rosaceana, but the species did not differ in responses to spinosad, acetamiprid, or B. thuringiensis (Table 1).

**Table 1.**
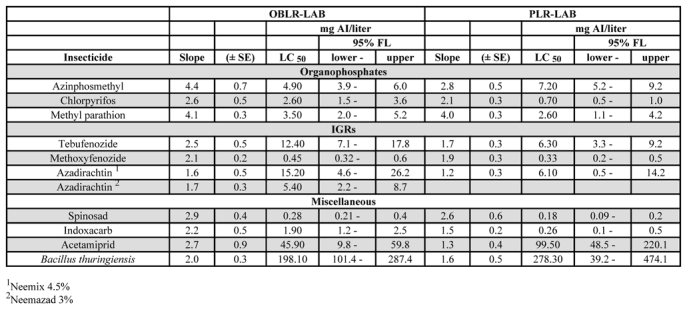
LC_50_ values of OP-susceptible laboratory colonies of OBLR and PLR to various insecticides, 1996–1997

In the 1996–97 samples, the first generation for both populations of field-collected C. rosaceana (MF1 and MA1) had significantly higher LC_50_ values to azinphosmethyl (9.2- to 10-fold) compared to the susceptible laboratory colony (Table 2). The field-collected populations had LC_50_ values for chlorpyrifos that did not differ from that of the laboratory colony and neither population was cross-resistant to azinphosmethyl (Table 2). The field populations were significantly more tolerant of tebufenozide compared to the laboratory colony. LCRs for methoxyfenozide (12.8- and 26.3-fold) were higher than those for tebufenozide, suggesting a higher degree of cross-resistance (Table 2). Tebufenozide and methoxyfenozide were cross-resistant to azinphosmethyl even though they had not been used againstC. rosaceana in the field. There was no difference in the tolerance between field populations of C. rosaceana to spinosad and the laboratory colony, but a weak (2-fold) negatively-correlated cross-resistance to spinosad was found in one C. rosaceana population (MA1) (Table 2).

**Table 2.**
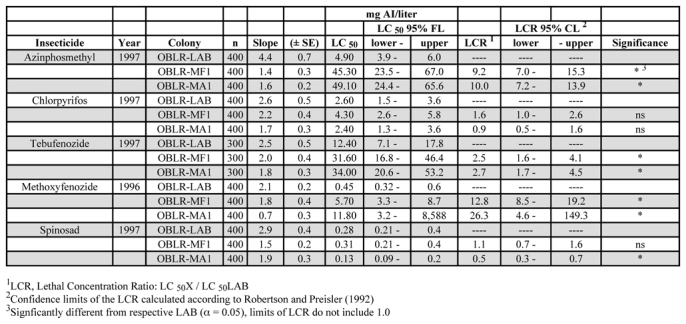
LC_50_ and LCR values of laboratory (susceptible) and G_1_ of field-collected populations of OBLR, 1996–97

All of the field (commercial orchard) populations of C. rosaceana were significantly resistant to azinphosmethyl relative to the laboratory colony (Table 3). Resistance levels were 5 to 27 fold, and resistance levels from the Mattawa and Milton-Freewater areas (MA and MF collections) were higher than found in 1997. The highest resistance levels were found in the Milton-Freewater area, which had a history of severe leafroller problems and liberal organophosphate use (J.F. Brunner unpublished data). In general, populations from the southern part of the region tended to be more resistant than those from the northern region (AR, BR).

**Table 3.**
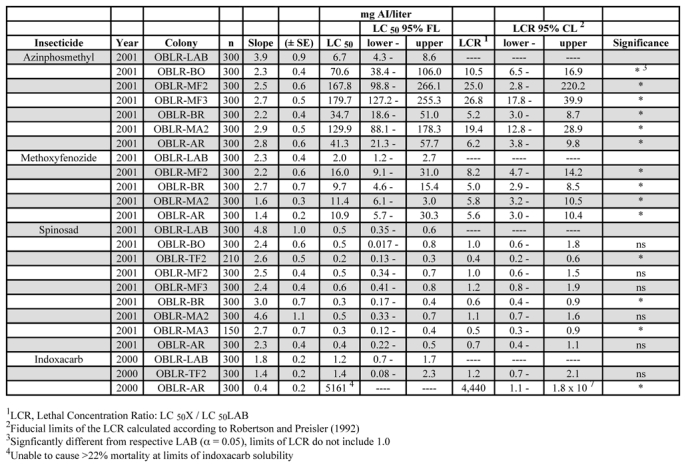
LC_50_ and LCR values for G_1_ of field-collected populations of OBLR in comparison to a susceptible laboratory population, 2000–01

All of the populations tested were resistant to methoxyfenozide and were also cross-resistant to azinphosmethyl (Table 3). However, there was a significant increase in the LC_50_ estimate for the laboratory population in 2001, 2.0 mg AI/liter, from 0.45 mg AI/liter in 1996. Thus, the LCR values did not increase in correspondence to the higher levels of azinphosmethyl resistance.

None of the azinphosmethyl-resistant field populations showed resistance to spinosad, with LC_50_ estimates ranging from 0.2 to 0.6 mg AI/liter (Table 3). Nevertheless, three of the eight populations had significantly different LCRs relative to the susceptible population. For these three populations, the LC_50_s were lower than the susceptible population, indicating the possibility of a negatively correlated cross-resistance, although the differences were small, similar to that found in 1997.

Only two C. rosaceana populations were tested with indoxacarb (Table 3). One, the TF2 population, was not significantly different the susceptible laboratory colony. The other, AR, had an extremely high LC_50_ estimate, and correspondingly high LCR. High mortality of larvae of this population could not be obtained with the rates used, resulting in a very flat slope for the probit line. While these are very preliminary data they confirm reports of high C. rosaceana resistance in Canada (Smirle *et al.* 2002) and are an indicator of potential problems for the use of this material against C. rosaceana. The AR population was only moderately resistant (6.2-fold) to azinphosmethyl.

Of the five P. pyrusana field populations tested, all were resistant to azinphosmethyl, including the TF1 and WV populations, which had not received any organophosphate insecticides for five years prior to collection (Table 4). Resistance levels ranged from 8.4- to 24.9-fold, with the highest level found in the CRO population.

**Table 4.**
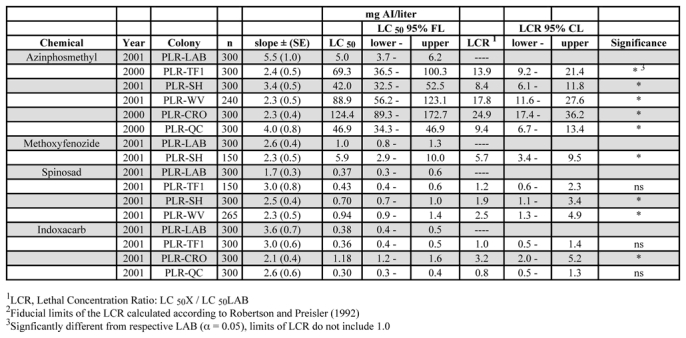
LC_50_ and LCR values for G_1_ of field-collected populations of PLR in comparison to a susceptible laboratory population, 2000–01

The only field-collected P. pyrusana population tested was resistant to methoxyfenozide, and was cross-resistant to azinphosmethyl (Table 4). Two of three field populations were slightly resistant to spinosad (1.9- to 2.5-fold) and they appeared to be cross-resistant to azinphosmethyl. The one population that was an exception was TF1, which had not received organophosphate insecticides for several years. Unlike C. rosaceana, all significant LCRs were positive for spinosad (>1). Response of P. pyrusana to indoxacarb was variable, with only one population having a significant LC_50_ higher than the laboratory colony and with an LCR greater than one. This is the same population (CRO) had the highest azinphosmethyl LC_50_.

## Discussion

Information on resistance levels and cross-resistance is an important factor in structuring a resistance management program for leafrollers. The prevalence of azinphosmethyl resistance in both leafroller species reflects the decades of exposure, and indicates its current unsuitability as a leafroller management tool. While cross-resistance would be likely with other organophosphate insecticides, the lack of cross-resistance to the organophosphate insecticide chlorpyrifos (1997) is consistent with the results of Dunley and Welter (2000), who found negatively-correlated cross-resistance in codling moth. The same would likely have been true of methyl parathion, which was relied on for leafroller control through the 1980's and also has been found to have a negative correlation to azinphosmethyl resistance in codling moth (Dunley and Welter 2000). Since methyl parathion is no longer registered for use in tree fruit and chlorpyrifos is restricted to prebloom use only, their negatively correlated cross-resistance is unavailable as tools in a resistance management program.

Cross-resistance between azinphosmethyl and the benzoylhydrazine insect growth regulators (tebufenozide and methoxyfenozide) means they must be used with caution, and only in a resistance management program. Such cross-resistance has been noted elsewhere (Sauphanor and Bouvier 1995; Carrière *et al.* 1996; Waldstein *et al.* 1999; Wearing 1998; Pree *et al.* 2001; Ahmad *et al*. 2002; Ahmad and Hollingworth 2004). The insect growth regulators are an important class of chemistry for use in mating-disruption based programs, and their conservation as tactics is vital. A clear demonstration of cross-resistance can provide convincing evidence to producers of the necessity of practicing resistance management (ffrench-Constant and Roush 1990). Too often there is a tendency for producers to make a one-for-one switch of insecticides after resistance becomes apparent placing greater selection pressure on the new product.

The insect pest management program for Washington apples has more tools for chemical control of key pests than at any time in the last 40 years. The variety of insecticide choices available to control apple pests makes apple integrated pest management decisions more complex, but also provides opportunities for reducing the historical reliance on broad-spectrum neurotoxic insecticides. Additionally, some of the new chemistries are just as effective as the organophosphates, but may provide more stability to apple integrated pest management due to selectivity and better conservation of biological control agents. This opportunity for improving integrated pest management could be squandered if new insecticides are not effectively managed by following good resistance management practices. The information presented here shows that new insecticides, such as methoxyfenozide, can face the potential of reduced efficacy even before they are introduced. While spinosad does not appear to have been influenced by some pre-existing cross-resistance to organophosphate insecticides in leafrollers, it is difficult to predict how long it will remain effective if over-used. While it might be possible to select a susceptible leafroller population for resistance to spinosad or some other new insecticide in the laboratory this requires a great deal of effort and the ability of such an approach to predict the rate of resistance development is unclear. Therefore it seems necessary to conduct surveys of field populations of leafrollers on a regular basis as a way of tracking changes in resistance levels.

Apple producers in Washington will need to adopt a new level of sophistication when it comes to managing new insecticides. While the newer insecticides generally have narrower spectrums of activity than older products, many still affect more than one pest. We are recommending to Washington apple producers that an insecticide, or insecticide class, not be used against the same pest in two consecutive generations. If a product is used to control a pest at one time in the growing season it would not be used later in the season (next generation) against the same pest. If the product is also effective against another pest it could be used unless the application timing would expose the first target pest to selection. For example, it is possible to apply a product like methoxyfenozide against codling moth in the spring and then against C. rosaceana in the summer. However, this latter application would also impact the second codling moth generation and thus be counter to resistance management practices. While new insecticides provide more choices for management of apple pests, strategies to preserve those choices will limit the options in order to provide long-term stability to integrated pest management programs.
